# Oncoplastic Breast Reduction: Maximizing Aesthetics and Surgical Margins

**DOI:** 10.1155/2012/907576

**Published:** 2012-11-13

**Authors:** Michelle Milee Chang, Tara Huston, Jeffrey Ascherman, Christine Rohde

**Affiliations:** ^1^Division of Plastic Surgery, Columbia University Medical Center, New York-Presbyterian Hospital, New York, NY 10032, USA; ^2^Plastic and Reconstructive Surgery Division, Stony Brook Medicine, Stony Brook, NY 11794, USA

## Abstract

Oncoplastic breast reduction combines oncologically sound concepts of cancer removal with aesthetically maximized approaches for breast reduction. Numerous incision patterns and types of pedicles can be used for purposes of oncoplastic reduction, each tailored for size and location of tumor. A team approach between reconstructive and breast surgeons produces positive long-term oncologic results as well as satisfactory cosmetic and functional outcomes, rendering oncoplastic breast reduction a favorable treatment option for certain patients with breast cancer.

## 1. Introduction

Surgeons who treat breast cancer strive to perform operations that are aesthetically pleasing without compromising oncologic outcome. Patients are more informed than ever and are encouraging their surgical teams to continue to evolve [1].

For treatment of their breast cancer, many women elect breast conservation therapy (BCT). BCT combines lumpectomy with postoperative radiation allowing a woman to preserve her breast. Factors leading to a greater use of BCT versus mastectomy include improved screening and earlier mammography which have resulted in an increased identification of small, early-stage breast cancers, an increased use of neoadjuvant chemotherapy which can shrink large tumors, and the patient's own preference to preserve her breast [2]. 

With breast preservation, cancer survival is affected by local control defined by appropriate clear margins. Despite a higher local recurrence rate, disease-free long-term survival is equivalent for patients undergoing total mastectomy and BCT. The premise of BCT involves both surgical excision and reconstruction, including an oncologically sound resection of the tumor, radiation of the resection bed, and preservation of the breast for enhanced aesthetic outcome [2].

To ensure clear margins of tumor resection in BCT, large volumes of breast tissue may need to be removed, leading to asymmetry, scarring, and deformity. Up to 30% of patients who have undergone BCT end up with a poor cosmetic outcome [3, 4]. Subsequent irradiation often then further compromises already suboptimal surgical results.

## 2. Oncoplastic Surgery

The initial reports of aesthetic techniques coupled with oncologic treatment were published in the 1990s [5]. The term “oncoplastic breast surgery” was coined in the mid-1990s [6]. Oncoplastic methods enable large tumor resections by marrying extirpative surgery with breast reduction surgery. Procedures are designed to anticipate and prevent unfavorable aesthetic outcomes, decreasing the rates to below 7% [7]. In addition, patients have the added benefit of a reduction mammaplasty, which may include a decrease in back, shoulder, and neck discomfort.

There are a number of oncologic advantages to oncoplastic breast reduction. A generous margin of tumor resection is feasible because a large volume of glandular tissue is removed [8, 9]. Furthermore, the resulting smaller breast size may improve the efficacy of radiation therapy. Lastly, reduction of the contralateral breast not only offers tissue sampling, but also theoretically reduces additional risk of breast cancer through removal of excess breast parenchyma [10]. The rate of occult breast cancers found in contralateral symmetrizing reduction specimens in patients undergoing breast reconstruction ranges from 4.6 to 11% [11–14].

Cosmesis in BCT (standard lumpectomy alone) is affected by breast size, with both very small- and very large-breasted women faring worst. Macromastia has been estimated in up to 40% of women treated with BCT [15]. In patients with macromastia, aesthetic outcome with lumpectomy alone might not be ideal. BCT in a large-breasted woman may leave an empty sac and can result in a ptotic breast, which can lead to a heterogeneous dose distribution. This is due to repeated positioning over an extended course of treatment [16]. Oncoplastic breast reduction has been found to circumvent these complications, relieving symptoms related to larger breasts, as well as treating the cancer itself [17].

Breast volume is important when considering oncoplastic surgery. Cochrane et al. has shown that as much as 10% of glandular breast volume can be removed without notable cosmetic deformities. In addition, the larger the breast is, the more tolerant it is to resection [18]. Delay and Clough demonstrated that up to 20% of breast volume can be excised, requiring local parenchymal rearrangement or skin excision for satisfactory results [19].

Communication between the breast and reconstructive surgeon is crucial. Preoperatively, this team approach is critical in defining areas of excision and in designing reduction techniques. The breast surgeon needs to be cognizant of breast aesthetics, volume, and symmetry, keeping in mind that referral to a plastic surgeon may be helpful. In turn, the reconstructive surgeon should understand oncologic surgical principles when creating a sound operative plan. 

## 3. Patient Evaluation and Counseling

Numerous factors are considered in patient selection. The most important selection criteria include (1) a patient's desire for smaller breasts and (2) the degree of the cancer surgeon's concern about aesthetic irregularities while resecting adequate specimen size. An ideal candidate requires a large-volume resection and has symptoms of macromastia (chronic headaches, back pain, neck pain, shoulder grooving, or intertriginous rashes). However, any patients with moderate-to-large sized breasts are still possible candidates for selection [10]. The oncoplastic procedure is applicable to either patients who have had no prior surgical intervention or those who have attempted breast conservation with positive margins.

A detailed history is critical. Symptoms of macromastia should be documented as well as factors that can impact wound healing or breast tissue perfusion such as: steroid use, smoking, diabetes, prior breast surgery, connective tissue diseases, or irradiation to the thorax. The presence of any of these factors should prompt further counseling regarding increased risk of complications such as fat necrosis, nipple necrosis, or other wound healing complications. Also, because a history of smoking predisposes to increased nipple and flap necrosis, measures for smoking succession must be pursued if the patient is currently smoking. A focused physical exam is also important. Height, weight, and body mass index should be recorded. An emphasis on breast size, shape, prior scars, degree of ptosis, and position of lesion is important. In addition, measurements of breast width, sternal notch-to-nipple distance, nipple-to-inframammary-fold distance, and NAC width may be taken. Asymmetries should be documented and made evident to the patient. Photographs should be taken for the medical record.

Breast measurements are important as an indicator of breast size, ptosis, and volume. They help to point out pre-operative asymmetries that may persist after surgery. There are no absolutes with breast measurements, but in general, patients with sternal notch-to-nipple (SN-N) distances of 35 or greater need to be counseled regarding the possibility of free nipple grafts. Greater SN-N distances risk poor perfusion to the nipple through the pedicle, leading to nipple/areola necrosis [20]. Patients for whom the SN-N distance will change by more than 10 cm are poorer candidates for vertical scar breast reductions because of the geometry of pedicle rotation within the skin reduction pattern.

After the decision for oncoplastic reduction has been made, the time course must then be considered. The immediate one-stage reconstruction approach is preferable, both for psychological and aesthetic reasons. Delayed reconstruction may be advisable for younger patients with extensive ductal carcinoma *in situ* (DCIS), as this group has a higher rate of positive margins. In such cases, preoperative counseling should be directed towards a two-stage procedure and reconstruction should be postponed until negative margins are confirmed [10]. 

Furthermore, both breasts should be integrated into the decision-making process and treatment plan. Immediate breast reconstruction on the contralateral breast is much more common, except in cases of patients with DCIS, as explained above. Symmetry is the most important factor for good cosmetic outcome. In order to spare additional surgery, surgeons will often reduce or symmetrize the contralateral breast in the first procedure. This, however, requires an educated approximation of the size and shape of the contralateral breast to the ipsilateral breast because it is impossible to know the final size of the ipsilateral breast following cancer ablation. Involution and edema of the breast following irradiation further exacerbates this situation. Following radiation, the treated breast will become firmer and often rise up on the chest wall. For this reason, some surgeons prefer to perform the contralateral symmetrizing reduction in a two-step delayed procedure. Fitoussi et al. showed a preferential shift from synchronous reconstruction to delayed contralateral symmetrizing reduction in 540 consecutive cases [21]. Despite these trends, studies show that immediate reconstruction is not only safe, but may also provide better aesthetic outcomes [22–24].

Patient counseling of possible complications, as well as the need for a total mastectomy (if margins are involved) is essential in the preoperative workup. After oncoplastic reduction, breast geometry is completely rearranged, potentially leaving margins unidentifiable. Patterns of recurrence can be significantly altered. Therefore, in our practice, if the margins are positive following this procedure, the necessary next step is usually a total mastectomy. In addition, decreased sensation, partial or total thickness skin loss, asymmetries, and wound-healing issues of the nipple are also complications that may arise from ablation, reduction, and subsequent radiation. 

## 4. Planning

 The two main surgical decisions that must be chosen when planning a breast reduction are the choice of incision and the type of pedicle on which the nipple areolar complex will be transposed. This is influenced by numerous elements. While tumor location is the most important factor, other considerations include previous scars/needle biopsy sites and whether these need to be excised, as well as the need for access to the axilla. While not always applicable, excision of skin overlying the tumor and its extent can also be included. The surgeon's comfort and preference for reduction mammaplastic techniques is an important factor as well. Lastly, thought must be given to the effect of radiation and the potential to change the eventual size of the breast. Radiation can lead to chronic edema and involution or shrinkage of the remaining breast tissue resulting in a smaller, firmer breast, which rides higher up on the chest wall.

## 5. Margin Evaluation

The most important goal of the oncoplastic approach is to resect the cancer with histologically negative margins. Oncoplastic breast reductions enable wider margins than standard lumpectomy alone. In our practice, to optimize positive margins, we try to remove the skin over the tumor whenever possible as well as the breast tissue and muscle fascia posterior to the tumor. Positive margins are associated with a significantly higher incidence of local recurrence [25, 26]. Intraoperative assessments of margins are advised, utilizing both pathologic specimen examination and radiologic imaging. Microcalcifications can be assessed via specimen mammogram with two 90-degree images. Intraoperative ultrasound use has shown a decrease in reexcision rate, especially in the cases with solid masses [27, 28].

 Histologic evaluation is also useful. Currently, frozen sections with touch preparation are one of the most accepted methods of intraoperative histologic assessment of margins. Other recently developed technologies, such as the Spectroscopy or MarginProbe (Dune Medical Devices, Caesarea, Israel), are now being evaluated for real-time intraoperative margin assessment [28].

 In our practice, we often mark the margins of resection with hemoclips. The clips serve as a guide to radiation oncologists for radiation therapy, especially in the delivery of an appropriate boost dose.

## 6. Skin Incision Types

**Figure 1:**
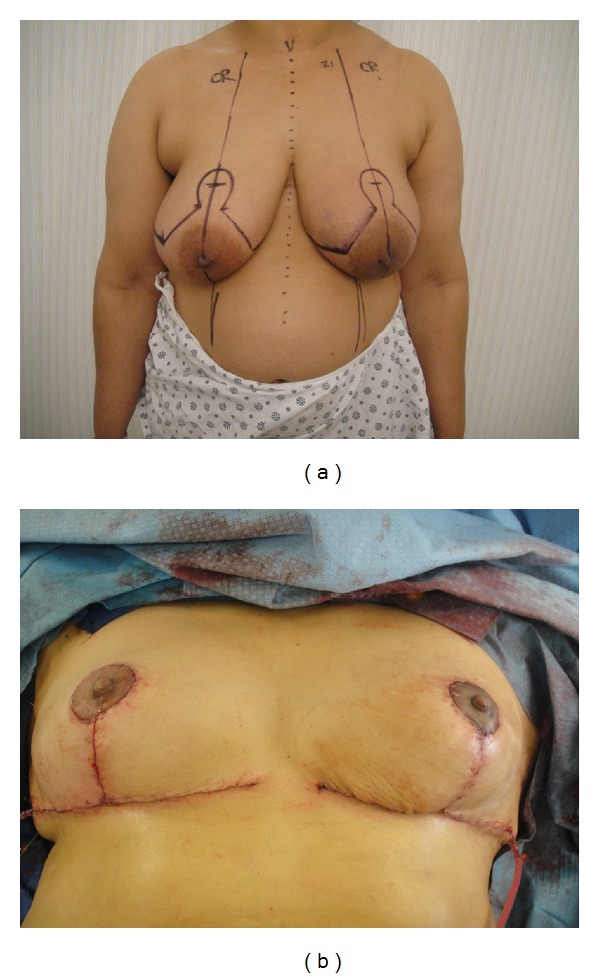
(a) Preoperative markings before Wise pattern superomedial pedicle oncoplastic breast reduction. The patient's breast cancer is located in the inferolateral breast. (b) Immediate on-table result after oncoplastic breast reduction showing location of scars.

 Following a preoperative evaluation and determination of timing, the next major decision to be made is the location and size of the incision. There are numerous incisions to choose from, each with their advantages and disadvantages. The Wise pattern (or inverted “T”) is the most commonly used incision for oncoplastic breast reductions because it offers the most opportunities for breast reshaping. This incision travels along the inframammary fold (IMF) and traverses up to the nipple (Figures 1(a) and 1(b)). The Wise pattern offers the surgeon much flexibility, with wide access to the breast parenchyma for use for a tumor in any location. This procedure also allows skin excision in both vertical and horizontal dimension and can be used with any pedicle. For lymph node sampling or clearance, a separate small incision may be needed in the axilla, depending on the pedicle and desire to avoid wide undermining.

 The vertical scar mammaplasty, first introduced by Lassus and modified by Lejour, is the second most commonly used incision for oncoplastic breast reduction [29, 30]. This incision is made around the nipple-areola complex (NAC) and extended down to the IMF. The vertical scar technique also allows good access to the breast parenchyma; breast skin reduction is accessible in the horizontal axis, and vertical size reduction is possible through cinching closure of the skin. One drawback of this technique is that the axilla is not easily reached. The classic Lejour breast reduction includes a reduction in breast volume via liposuction. This step is not advisable in cancer operations because of the risk of seeding tumor cells.

 The omegaplasty (or bat-wing incision) traverses above and alongside the nipple. While not cosmetically ideal, it does provide good access to superomedially located tumors [31]. Omegaplasty requires no undermining (which is better for radiation), but fails to decrease breast volume or address ptosis. This approach can also result in pseudoptosis if there is too much skin or parenchyma resected with an incision superior to the nipple.

The lateral mammaplasty incision runs from the nipple out laterally toward the anterior axillary line. It may also be extended superiorly to gain access to the axilla. Because most tumors are laterally located, this incision's lateral access makes it an important approach. One advantage of the lateral mammaplasty is the avoidance of thin, large surface area dermal flaps, creating thick flaps that are more tolerant to radiation. Medial mammaplasty incisions are the reverse of the lateral mammaplasty, traveling toward the sternum. This technique it the most useful for medially located tumors [32].

 The periareolar approach is not as common because it does not permit a great deal of skin excision or volume reduction. The periareolar incision is solely around the nipple and does not extend out onto the breast. Still, this procedure has good access to the upper pole of the breast. Women with mild ptosis and for whom a mastopexy might be considered are good candidates for the periareolar technique.

## 7. Pedicle Options

 After the location and size of incision has been determined, and the extent of tumor resection has been defined, a decision must be made for the origin of the dermoglandular pedicle on which the vascular and nervous supply to the nipple will be carried. The pedicle is important not only to achieve a satisfactory aesthetic outcome, but also to preserve the blood and nerve supply to the NAC. There is a myriad of pedicles, all of which have their cosmetic and sensory advantages or disadvantages. In oncoplastic breast reduction surgery, the pedicle is chosen based on what remains after the ablation of the tumor. For example, a lower-pole tumor will require resection of glandular tissue in the lower half of the breast, leaving the reconstructive surgeon to choose between various superior pedicles. The pedicles most commonly used are superior, inferior, and medial.

 The superior pedicle is preferred for being solid, reliable, and better able to preserve nipple sensation. Limitations of this pedicle arise from difficulty in moving the nipple long distances, especially in patients with significant hypertrophy of the breast. Use of this pedicle may be difficult with large reductions, where molding may result in only superior fullness. Superior pedicles are best for lower-quadrant tumors, particularly in moderate-sized breasts. 

**Figure 2:**
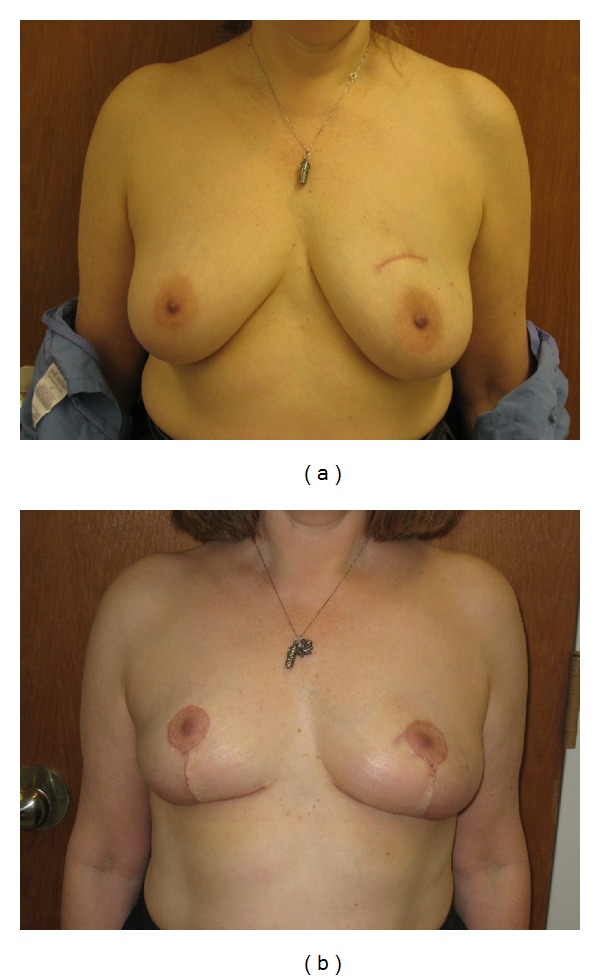
(a) Preoperative appearance of patient with left breast cancer. (b) Two-month postoperative appearance of patient after inferior pedicle Wise pattern breast reduction. Note incorporation of lumpectomy scar over superomedially located tumor into the incision for the areola.

 Inferior pedicles are reliable for tumors in any position. One caution to take is that it lacks parenchymal support and breasts may eventually sag or “bottom out,” resulting in excess skin and tissue between the nipple and IMF. This technique is ideal for larger breasts with longer sternal notch-to-nipple distances as well as tumors located in the upper quadrants of the breast (Figures 2(a) and 2(b)).

 The lateral pedicle is an option for medial tumors that extend into the upper or lower quadrants. This pedicle is not frequently used cosmetically because if the pedicle is too thick, the breast will be too full laterally. It is normally reserved for women with small-to-moderate-sized breasts and requires a mastopexy or a minor reduction.

**Figure 3:**
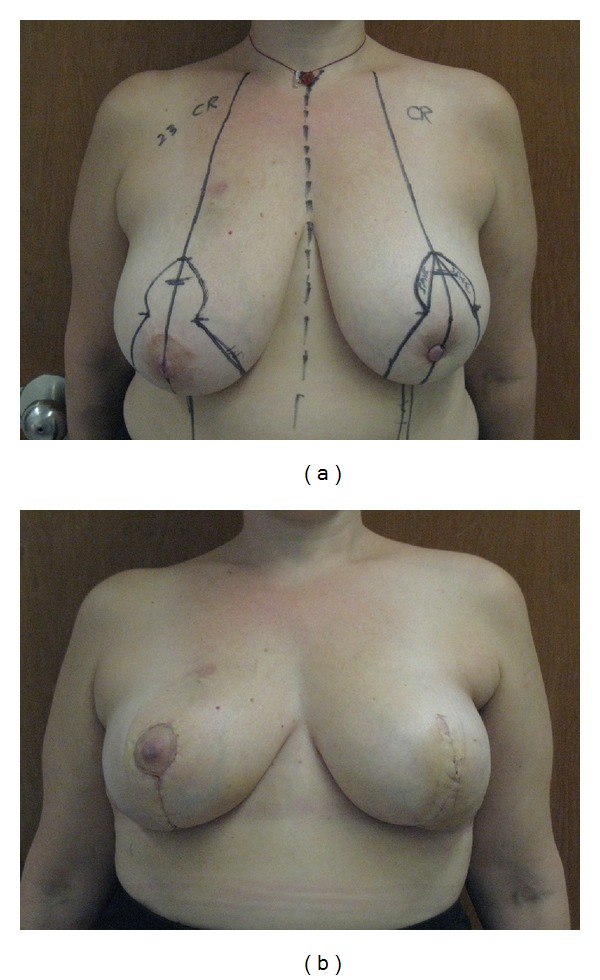
(a) Preoperative markings for Wise pattern oncoplastic breast reduction with planned excision of the left nipple areola complex. (b) Two-week postoperative result showing bilateral oncoplastic breast reductions with excision of left nipple-areola complex. Patient will then have nipple-areola reconstruction after completion of radiation.

 Free-nipple grafting during reduction mammaplasty is most applicable for patients with gigantomastia. In such cases, preservation of blood supply and nerves to the nipple is limited by the length of the pedicle needed to carry the NAC into its new position and by the ability to reduce the breast with a large pedicle. The nipple-areola complex is removed as a skin graft, the breasts are reduced, and the NAC is then sutured in the appropriate position on the breasts. Nipple sensation is lost with this procedure, and hypopigmentation results, usually taking at least one year for pigmentation to return. In cases where oncologic margins require removal of the nipple-areola complex, it can be excised without negatively impacting breast shape, as long as adequate skin is preserved (Figures 3(a) and 3(b)). The nipple and areola can then be reconstructed after completion of radiation, using any standard method.

## 8. Postoperative Radiation

 Radiation therapy is the second phase of BCT, starting 3 to 6 weeks after the reduction procedure once the incisions have healed. Therapy includes whole-breast irradiation, as well as a boost to the tumor bed to kill any residual microscopic deposit of cells that surgery may have missed. Cosmetically, radiation also tends to diminish scarring on the breast [33]. During surgery, surgeons should be mindful of imminent radiation by avoiding extensive skin-gland dissection and avoiding excessively long parenchymal pedicles that may be compromised and predisposed to fat necrosis. Patients should be informed that radiation therapy can result in chronic edema of the irradiated breast, or contraction and scarring, such that initial postoperative symmetry can be permanently affected. 

## 9. Complications

 In the literature, the complication rate for oncoplastic bilateral breast reduction ranges between 17% and 24% [7, 10, 34]. Common complications include skin necrosis, infection, partial or complete nipple areolar complex necrosis, and suture-line dehiscence. Like reduction mammaplasty patients without cancer, obese patients and regular smokers suffer from higher complication rates postoperatively.

 If adjuvant chemotherapy is planned, it may begin once healing of the incisions has occurred and can be followed by radiation therapy. Complications that interfere with wound healing may delay the onset of chemotherapy or radiation therapy.

## 10. Oncologic Results

 Fitoussi conducted the largest study to date, following 540 patients who underwent oncoplastic breast surgery for cancer, with a median followup of 49 months. Close or positive margins occurred in 18.9%, with subsequent mastectomy being necessary in 9.4%. At five years, 90.3% reported a satisfactory aesthetic outcome. Five year overall and distant disease-free survival rates were 92.9% and 87.9% respectively, with local recurrence in 6.8% [21]. When compared to the standard BCT, comparable values have been found, demonstrating the equivalent oncologic safety between the two. Rietjens followed 148 women for a median 74 months to report a 3% rate of local recurrence [35]. Kayar recorded 116 patients over a period of 10 years, demonstrating overall survival rates at 100%, 89.1%, and 53.8% for stage I, stage II, and stage III, respectively [36].

 Chakravorty et al. compared outcomes from 150 cases that had utilized the oncoplastic conservation techniques (77 of which were for oncoplastic reduction) with 440 cases, which used standard breast conserving surgery. At a 28-month followup and a subsequent projected 6-year local recurrence rate, oncoplastic breast conserving techniques were found to decrease reexcision rates, with oncological outcomes similar to that of standard breast conservation [37]. This finding of decreased reexcision rates is expected given the increased volume of tissue that can be removed with oncoplastic breast reduction and the need for mastectomy if there are positive margins.

## 11. Oncologic Surveillance

 Less-experienced breast radiotherapists and radiologists may find the complex glandular reshaping from oncoplastic reduction techniques more challenging to examine on mammograms. Women with oncoplastic reductions are more likely to have a greater number of postoperative mammograms and ultrasounds as well as a greater rate of tissue sampling compared to women who have undergone partial breast reconstruction [38].

 Oncoplastic breast reduction does not appear to affect cancer screening for recurrence. Although scar tissue, epidermal inclusion cysts, or fat necrosis may appear suspicious on physical exam, mammogram, ultrasound, or MRI, evaluation can be done with fine needle aspiration or core needle biopsy [39]. Typically, in postoperative healing, fat necrosis will present early on and slowly resolve with time, with complete or incomplete resorption. Because of such situations, each follow-up visit should be with the same oncologic surgeon. Mammographic findings from a study by Mendelsohn et al. found scarring and fibrosis in 50% of patients, fluid accumulations in 40% of patients, and dystrophic calcifications in 10% of patients [39]. Even though cancer screening is not compromised, patients who undergo oncoplastic reduction require more postoperative tissue sampling than those who receive traditional BCT [10].

## 12. Oncoplastic Outcomes

 Currently, a widely accepted objective study for investigating cosmetic outcomes is not available. The BREAST-Q is a validated data set tool that may bring more insight into this matter. Through pre- and postoperative questionnaires, the BREAST-Q quantifies patient satisfaction and health-related quality of life experience in a psychometrically sound and clinically meaningful manner [40]. Patients report significantly improved body image, functional quality of life, and cosmesis when treated with BCT versus radical mastectomy [41]. More specifically for oncoplastic surgery, available publications indicate an overall satisfaction in treated patients. Chang et al. collected surveys from 20 patients with 70% rating the cosmetic outcome as excellent and 100% reported a high degree of satisfaction with cosmetic and functional results [42]. Goffman et al. established a panel, which included a surgical oncologist, an oncology nurse, a radiation oncologist, and a patient to evaluate cosmetic and functional results. Out of 55 patients, 72% evaluations gave excellent and very good marks [43]. Lastly, in a study conducted by Losken et al. 95% of women reported satisfactory aesthetic results after a six month followup [10].

## 13. Authors' Experience and Technique

All patients who the breast surgeons feel will have a significant deformity following lumpectomy are referred to a plastic surgeon. Small-breasted women generally decide to undergo mastectomy and reconstruction. For women with moderate-to-large sized breasts, there is an extensive discussion regarding relative advantages and disadvantages to an oncoplastic breast reduction versus mastectomy and reconstruction (as detailed in the rest of this paper). 

Once the decision is made for oncoplastic breast reduction, a combined operation is scheduled. If wire localization is required, two wires are generally used at each tumor site to precisely localize the cancer within the breast. If the patient has had a prior lumpectomy, all efforts are made to incorporate the lumpectomy scar within the skin incision. Skin markings are made to include prior scars and biopsy sites whenever possible (Figure 2). This can sometimes mean adjusting markings more superiorly, laterally, or medially. Patients are counseled that the new position of the nipple-areola complex can be affected by these adjustments away from their ideal position at the breast meridian, inframammary fold, or near the midhumeral line. The choice of Wise pattern or vertical skin markings is made based on breast size, degree of ptosis, location of tumor, location of prior scars, and sternal notch-to-nipple distance. The Wise pattern incorporates a larger skin excision area. It is therefore more useful for incorporation of prior scars or skin over the tumors and is used more often in oncoplastic breast reductions. 

The plastic surgeon and breast surgeon perform the lumpectomy together in order to maximize margins and aesthetics. The plastic surgeon starts the operation, making the incisions and creating the pedicle, with the guidance of the oncologic surgeon. When possible, the skin over the tumor is included in the specimen. As the wires or lumpectomy cavity are approached, the breast surgeon takes over to excise around the tumor. Posteriorly, the breast tissue is removed deep to the tumor, including muscle fascia, in order to maximize the posterior margin. The corresponding author prefers to use a superomedial pedicle whenever tumor location permits (personal preference), although the operation is similar with any pedicle. The reduction proceeds in a standard fashion [44]. The entire breast reduction specimen is removed as a single specimen incorporating the lumpectomy specimen, in order to avoid cutting across a margin. Once the specimen is removed, the breast surgeon reevaluates the remaining breast and removes any additional margins deemed necessary. Breast closure then proceeds in a standard fashion, with rotation of the superomedial pedicle into the keyhole and closure of the lateral and medial pillars. This technique enables the removal of multiple lumpectomy specimens, even the ones in completely different areas of the breast, since a wide area of skin and breast is removed. The plastic surgeon does not hesitate to remove more tissue than required by a standard breast reduction, in order to provide the needed oncologic margins. 

To make up for the changes in geometry, additional tissue rearrangement within the breast may be necessary to provide the best shape and symmetry. Consequently, we advise all patients that a mastectomy would be needed if the margins return positive. Theoretically, a reexcision can be attempted depending on the original location of the tumor in relation to the reduction, and the location of the positive margin. Alternatively, the patient and oncologist may decide to give a radiation boost to the involved breast. However, we generally do not advise patients that a reexcision is likely possible, and we prepare them for the possibility of mastectomy if the margins are involved. 

Over the last 4 years, we have performed over 30 oncoplastic breast reductions. There has been one positive margin at a nipple and the patient ended up with a mastectomy on that side. No other patients were reported with involved or close margins. Although we have performed a relatively smaller number of oncoplastic breast reductions compared with mastectomy reconstructions, our rate of 3.33% positive margins compares favorably with published rates of positive margins (11-12%) after lumpectomy [45, 46]. As discussed preoperatively, the patient presenting with positive margins ended up with a mastectomy. In retrospect, if there was high suspicion for involvement of the nipple with cancer, she could have had a breast reduction with central breast removal and later nipple reconstruction, as the patient in Figure 3 had. In an analysis of 540 cases, close or involved margins occurred in 18.9 percent, with mastectomy being necessary in 9.4 percent [17]. We postulate that our rate of positive and close margins is less than 18.9% because our techniques involve removing skin over the tumor site and/or fascia over the muscle, and we tend to use techniques that remove a large amount of surrounding tissue. Fittousi et al. described their experience with a variety of “aesthetic” and “combination” techniques for oncoplastic breast surgery, and it is not clear how many were specifically oncoplastic breast reductions [21]. The authors remark that one-half of the patients with involved margins were “satisfactorily managed oncologically with either repeated oncoplastic breast surgery or radiotherapy boost” [17]. The authors do not elaborate as to whether the patients who had repeat oncoplastic breast surgery initially had a reduction pattern surgery, or if they initially had a more limited tissue rearrangement that enabled repeat excision. In our practice, we counsel oncoplastic breast reduction patients that a mastectomy would likely be the next step if a margin is involved, but, of course, any case would be evaluated individually.

This issue of mastectomy if there is a positive margin would seem to argue against oncoplastic breast reductions, since patients usually have a chance at reexcision with lumpectomy alone. By agreeing to an oncoplastic breast reduction, they would seem to be agreeing to a single attempt at a lumpectomy only. However, for most patients in our practice choosing this procedure, the decision is not between a standard lumpectomy or oncoplastic breast reductions; it is a choice between oncoplastic breast reductions or mastectomy. Patients deemed candidates for oncoplastic breast reductions are those for whom standard lumpectomies would be too deforming (because of breast size, tumor size, multiple tumors, or tumor location) or those with symptomatic macromastia who desire breast reduction for the added symptom relief. Therefore, they are willing to try oncoplastic breast reductions as an alternative to mastectomy. Additionally, the rate of positive margins is much lower than that with standard lumpectomies, so it is rare that these patients do indeed go on to need a mastectomy.

## 14. Conclusions

 In some parts of the United States, a potential lack of available reconstructive plastic surgeons limits combined treatment. Breast surgeons are then left with the choice of either referring their patients to larger centers or attempting to learn the reconstructive procedures themselves [47]. Despite these limitations, ideally, a combined approach with a breast surgeon and plastic surgeon provides the best results for the patient.

 Management of patients with breast cancer is also changing. Surgeons are constantly looking for new, less invasive, and more cosmetically favorable techniques to help patients manage their disease and live with the results of their treatment.
